# Distinctive Roles of YAP and TAZ in Human Endothelial Progenitor Cells Growth and Functions

**DOI:** 10.3390/biomedicines10010147

**Published:** 2022-01-11

**Authors:** Phatchanat Klaihmon, Chanchao Lorthongpanich, Pakpoom Kheolamai, Sudjit Luanpitpong, Surapol Issaragrisil

**Affiliations:** 1 Siriraj Center of Excellence for Stem Cell Research, Faculty of Medicine Siriraj Hospital, Mahidol University, Bangkok 10700, Thailand; phatchanat.kla@mahidol.ac.th (P.K.); sudjit.lua@mahidol.ac.th (S.L.); surapol.iss@mahidol.ac.th (S.I.); 2 Center of Excellence in Stem Cell Research and Innovation, Faculty of Medicine, Thammasat University, Pathum Thani 12120, Thailand; kpakpoom@staff.tu.ac.th

**Keywords:** hippo signaling pathway, endothelial progenitor cells, YAP/TAZ

## Abstract

The hippo signaling pathway plays an essential role in controlling organ size and balancing tissue homeostasis. Its two main effectors, yes-associated protein (YAP) and WW domain-containing transcription regulator 1, WWTR1 or TAZ, have also been shown to regulate endothelial cell functions and angiogenesis. In this study, the functions of YAP and TAZ in human endothelial progenitor cells (EPCs) were investigated by a loss-of-function study using CRISPR/Cas9-mediated gene knockdown (KD). Depletion of either YAP or TAZ reduced EPC survival and impaired many of their critical functions, including migration, invasion, vessel-formation, and expression of pro-angiogenic genes. Notably, TAZ-KD EPCs exhibited more severe phenotypes in comparison to YAP-KD EPCs. Moreover, the conditioned medium derived from TAZ-KD EPCs reduced the survivability of human lung cancer cells and increased their sensitivity to chemotherapeutic agents. The overexpression of either wild-type or constitutively active TAZ rescued the impaired phenotypes of TAZ-KD EPCs and restored the expression of pro-angiogenic genes in those EPCs. In summary, we demonstrate the crucial role of Hippo signaling components, YAP and TAZ, in controlling several aspects of EPC functions that can potentially be used as a drug target to enhance EPC functions in patients.

## 1. Introduction

In addition to mature endothelial cells that line the inner surface of the blood vessel wall, the bone marrow-derived circulating endothelial progenitor cells (EPCs), have been shown to play essential roles during postnatal tissue neovascularization under both physiological and pathological circumstances [1,2,3]. Neovascularization requires several EPC functions, including their ability to mobilize from blood circulation into the target tissues before extensively proliferating, differentiating to mature endothelial cells, and generating new vascular structures [4]. Moreover, EPCs also help maintain vascular integrity by releasing numerous pro-angiogenic factors that enhance the survival and functions of normal endothelial cells [5].

The hippo signaling pathway is an evolutionarily conserved master regulator of several tissue functions, including cell proliferation, regulation of organ size, and, recently, vascular homeostasis and neovascularization. The hippo signaling pathway consists of a series of core kinases that regulate their downstream targets. Yes-associated protein (YAP) and transcriptional co-activator with PDZ-binding motif TAZ (also known as WWTR1) are critical mediators of the Hippo signaling pathway. YAP and TAZ function as transcriptional co-factors which, in association with several DNA-binding partners such as TEA Domain Transcription Factor 1 (TEAD1), Runt-related transcription factor (RUNX), Tumor Protein P73 (p73), Suppressor of Mothers against Decapentaplegic (SMAD), T-Box Transcription Factor 5 (TBX5), Early growth response protein 1 (ERG-1), and Erb-B2 Receptor Tyrosine Kinase 4 (ERBB4), regulate the expression of their target genes [6]. The activity of YAP and TAZ is inhibited by the large tumor suppressor (LATS) kinases which phosphorylate YAP and TAZ at specific serine residues and prevent their nuclear translocation. Although there is evidence showing that the Hippo signaling pathway controls the survival and activity of mature endothelial cells [7,8,9], the regulatory roles of YAP and TAZ in EPC functions are still largely unknown.

Moreover, several previous reports have demonstrated the roles of YAP [10,11,12,13,14,15] and TAZ [16,17,18] in mature endothelial cells and the angiogenic process. Even though YAP and TAZ possess less than 60% amino acid sequence similarity, they are often considered to control the same group of target genes and have similar functions [19,20].

In the present study, we, therefore, investigated the roles of YAP and TAZ in the regulation of various EPC functions, including survivability, migration, invasion, vessel formation, and the expression of pro-angiogenic genes by utilizing genetic manipulation and pharmacological treatment. We found that loss of TAZ profoundly impaired EPC functions and could sensitize human lung cancer cells to apoptosis.

## 2. Materials and Methods

### 2.1. Cell Culture

This study was approved by the Ethics Committee of the Faculty of Medicine Siriraj Hospital, Mahidol University (COA no. Si 112/2020). EPCs were isolated from cord blood mononuclear cells of healthy donors and pooled together to generate one EPC cell line to minimize the biological variations between donors. EPC cells were grown on 10 μg/mL fibronectin-coated vessels in EndoGRO-LS complete medium (Millipore, MA, USA) supplemented with 10% fetal bovine serum (FBS), 100 U/mL Penicillin, and 100 μg/mL Streptomycin. National Cancer Institute lung cancer cell lines, NCI-H292 and A549, were obtained from American Type Culture Collection (ATCC) (Manassas, VA, USA) and cultured in RPMI1640 medium containing 10% FBS and antibiotics. All cells were maintained in a humidified atmosphere of 5% CO_2_ environment at 37 °C.

### 2.2. Generation of Knockdown Cells

All-in-one lentiviral plasmids carrying SpCas9-puromycin resistance and gRNA sequence against human *TAZ* (*pLV(CRISPR)-hCas9:T2A:Neo*-*U6 > hWWTR1* (gRNA#5147)) and *YAP* (pLV(CRISPR)-hCas9:T2A:NeoU6 > hYAP1 (gRNA#211)) were purchased from VectorBuilder (Chicago, IL, USA). Lentivirus production was performed using HEK293FT packaging cell (ATCC) in conjunction with pCMV.dR8.2 dvpr lentiviral packaging and pCMV-VSV-G envelope plasmids (Addgene #8485 and 8455) [21]. EPCs were incubated with lentiviral particles in the presence of hexadimethrine bromide for 24 h, and the transfected cells were treated with 3 μg/mL puromycin for 3 days and analyzed before use by Western blotting.

### 2.3. Cell Migration and Invasion Assays

Cell migration was conducted by wound healing assay. A monolayer of cells was cultured in 24-well plated (Corning, NY, USA) and then a wound space was created with a 1-mm tip width and allowed to migrate for 24 h. Photographs were taken under a phase-contrast microscope, and wound spaces were determined. Transwell invasion assay was performed using a 24-well plate unit with polycarbonate filters (8-μm pore size) (Corning) pre-coated with Matrigel (BD Biosciences, San Jose, CA, USA). Without Matrigel coating, the inserts were used for migration assay as an alternative to the scratch wound assay. Filling the lower chamber with 10% FBS-containing medium, tested cells were seeded into the upper chamber in a 2% FBS-containing medium and incubated for 48 h. Non-migrating/invading cells were removed from the upper side of the membrane using a cotton swab, and migrated/invaded cells were stained with 10 μg/mL Hoechst 33,342 dye (Thermo Scientific, MA, USA) for 30 min. Inserts were visualized and scored under a fluorescence microscope (Eclipse Ti-U with NiS-Elements, Nikon, Tokyo, Japan).

### 2.4. Senescence Assay

According to the manufacturer’s instructions, cellular senescence was detected using a senescence-associated β-galactosidase (SA-β-gal) staining kit (Abcam, Cambridge, UK).

### 2.5. Vessel-Formation Assay

EPCs at 3 × 10^4^ cells were plated on a Matrigel-coated 24-well plate in a complete medium. After 12 h of culture, the vessel formation derived from EPCs was photographed, and the tube area was quantified by ImageJ software (Bethesda, MA, USA).

### 2.6. Detection of Side Population (SP)

Lung cancer cells were labeled with 5 μg/mL Hoechst 33,342 in DMEM medium containing 2% FBS in the presence or absence of 25 μM ABCG2 inhibitor fumitremorgin C (FTC; Torcris, Ellisville, MO, USA) at 37 °C for 90 min. SP analysis was performed using BD FACS Aris Fusion cell sorter equipped with a near UV laser and Hoechst Blue and Red filters. SP fraction was calculated based on the disappearance of SP cells in the presence of FTC.

### 2.7. Analysis of Apoptosis and Cell Cycle Distribution

Cells were stained with PE-conjugated annexin-V and 7-aminoactinomycin D (7-ADD) (BD Biosciences; San Jose, CA, USA) in the binding buffer in the presence of Ca^2^^+^ for 15 min while BD CycleTest™ Plus DNA reagent kit (BD Biosciences) was used in the detection of DNA distribution in cell cycle assay according to the manufacturer’s instruction. Samples were subjected to FACS analysis by FACS Canto cytometer (BD Biosciences).

### 2.8. Gene Expression Analysis by RT-qPCR

Total RNA was extracted using TRIzol reagent (Invitrogen, Carlsbad, CA, USA). cDNA was prepared using 2 μg of RNA and reverse transcribed using RevertAid First Strand cDNA Synthesis kit (Thermo Fisher, Waltham, MA, USA). qPCR assay was carried out in a final volume of 10 μL using SYBR™Green PCR master mix (Applied Biosystems, Foster City, CA, USA) in a CFX384 Touch Real-Time PCR Detection System (Bio-Rad Laboratories, Hercules, CA, USA) using the following protocol: 95 °C initial denaturation for 10 min, followed by 40 cycles of denaturation (95 °C, 10 s), annealing (60 °C, 10 s), and extension (72 °C, 40 s). The relative quantity of a target gene was calculated by normalization with *GAPDH*. Primer sequences are listed in Table 1.

### 2.9. Western Blotting

Cell pellets were incubated in a commercial lysis buffer (Cell Signaling Technology, Danvers, MA, USA) and a protease inhibitor mixture (Roche Molecular Biochemicals, Indianapolis, IN, USA) at 4 °C for 30 min. Protein content was analyzed using BCA protein assay (Pierce Biotechnology, Rockford, IL, USA) and equal amounts of proteins were resolved under denaturing conditions by SDS–PAGE and transferred onto PVDF membranes (Merck Millipore, Burlington, MA, USA). Membranes were blocked with 5% nonfat dry milk, incubated with appropriate primary antibodies at 4 °C overnight, and subsequently incubated with peroxidase-conjugated secondary antibodies for 1 h at room temperature. The immune complexes were analyzed by enhanced chemiluminescence detection system on a digital imager (ImageQuant LAS, GE Healthcare, Pittsburgh, PA, USA).

### 2.10. Statistical Analysis

Data were presented as the mean ± standard derivation. The significance of the differences between groups was determined via the non-parametric Kruskal-Wallis test, and *p* < 0.05 was considered statistically significant.

## 3. Results

### 3.1. Dobutamine Inhibited the Hippo Signaling Pathway and Down-Regulated Their Endothelial-Related Target Genes

**Figure 1:**
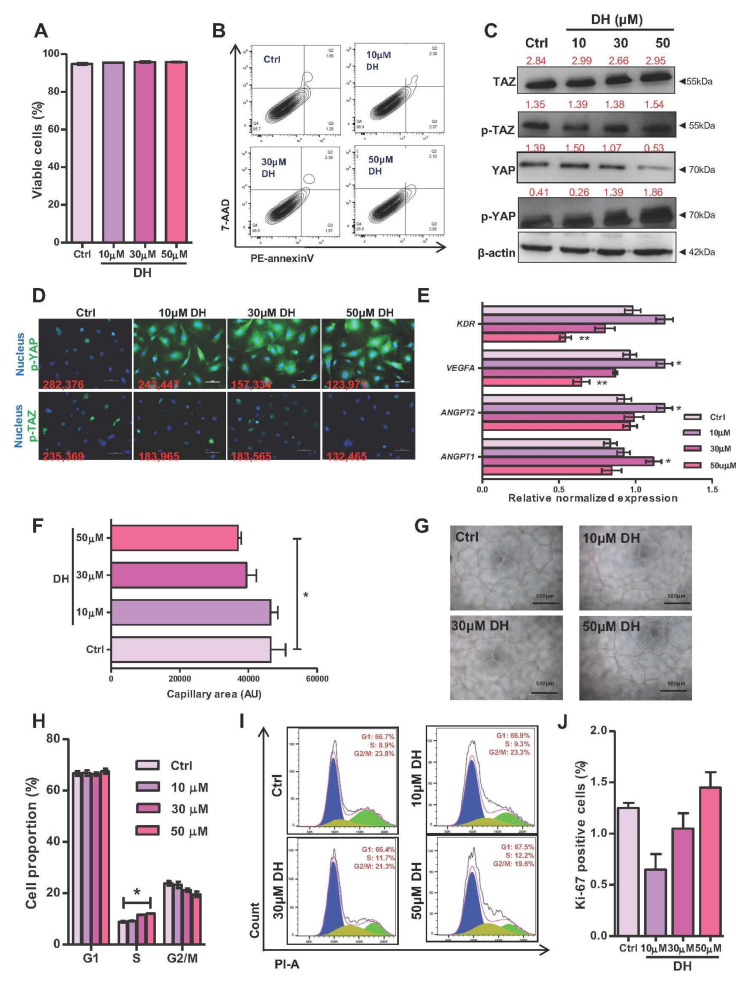
Effects of DH on human EPCs. EPCs were treated with DH at concentration of 0–50 μM for 24 h, then cells were analyzed for apoptosis detection by flow cytometric analysis (**A**,**B**). Protein levels of Hippo components; YAP/TAZ and their phosphorylated forms of DH-treated EPCs were determined by Western blotting and relative levels to β-actin were labeled in red (**C**). Localization of phosphorylated YAP and TAZ were addressed by immunofluorescence staining and fluorescent intensity of nuclear YAP and TAZ were labeled red (**D**). mRNA levels of candidate angiogenic genes in DH-treated EPCs were quantified by RT-qPCR (**E**). EPCs were subjected to tube formation assay in the presence of DH at indicated concentration and capillary area were measured after 12 h (**F**,**G**). Cell cycle analysis of DH-treated EPCs were measured by propidium iodide-staining DNA content and analyzed by flow cytometry (**H**,**I**). Proliferation assay by Ki-67 staining of DH-treated EPCs as determined by FACS analysis (**J**). All experiments were performed at least three times independently with technical duplicate and data were expressed as mean ± SEM. Scale bar 500 μm. * *p*-value < 0.05; ** *p*-value < 0.01.

Dobutamine (DH) is one of the Hippo signaling pathway inhibitors which alter the expression of YAP target genes by preventing the nuclear translocation of YAP [22]. We initially determined the cytotoxic effect of DH on human EPCs and found that DH, even at the highest concentration examined (50 µM), did not significantly affect EPC viability (Figure 1A,B). DH treatment increased the level of phosphorylated YAP and reduced the level of active YAP protein in EPCs in a concentration-dependent manner. In contrary to its effect on YAP, DH did not affect the level of phosphorylated-TAZ and active TAZ proteins in EPCs (Figure 1C). To confirm this, the Immunoblotting of nucleo-cytoplasmic fraction of DH-treated EPCs were provided in Supplementary Figure S1. The immunofluorescent staining also confirmed the cytoplasmic retention of phosphorylated YAP in EPCs treated with DH (Figure 1D). These results suggest that DH inhibits Hippo signaling in EPCs by increasing YAP phosphorylation and, by doing so, preventing its nuclear translocation. We next investigated whether the reduction of YAP could result in the differential expression of endothelial genes, VEGFA, KDR, ANGPT2, and ANGPT1, in EPCs. The results showed that DH significantly down-regulated both VEGFA and KDR (VEGF receptor) gene expression levels in a dose-dependent manner (Figure 1E). Moreover, the higher concentration of DH impaired the ability of EPCs to form capillary-like structures on Matrigel (Figure 1F,G). DNA distribution analysis also revealed the cell cycle arrest of DH-treated EPCs at the S-phase (Figure 1H,I). In addition, Ki-67 staining assay revealed that this cell cycle arrest caused by DH did not result from defective proliferation capacity (Figure 1J).

### 3.2. Phenotypic Characteristics of TAZ and YAP-Depleted EPCs

**Figure 2:**
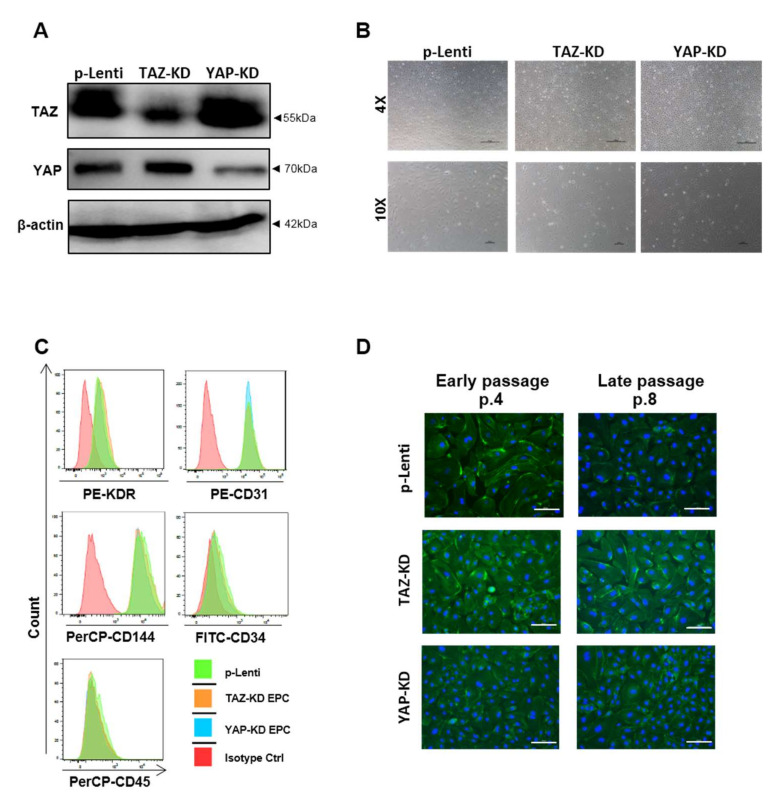
Phenotypic characteristics of YAP and TAZ-knockdown EPCs. Western blotting confirmed the CRISPR/Cas9-mediated knockdown of YAP and TAZ in EPCs (**A**). Phase-contrast microscopic photos of KD cells were illustrated at 4X and 10X magnification (**B**). A representative histogram from FACS analysis of typical surface markers of EPCs, including positivity of CD34, CD31, CD144, KDR, and negativity of CD45, was shown (**C**). Immunofluorescent staining of phalloidin (F-actin; green) for detecting cytoskeletal alignment and nuclear staining by DAPI (blue) in EPCs at early and late passage were presented at 20× magnification (**D**). Scale bar = 100 μm.

To further determine the roles of the Hippo signaling pathway in EPCs, the *YAP* knockdown (YAP-KD) and *TAZ* knockdown (TAZ-KD) EPCs, which are depleted of YAP and TAZ proteins, have been established using CRISPR-Cas9 gene editing. YAP-KD EPCs expressed a lower level of YAP and normal level of TAZ, while TAZ-KD EPCs expressed a lower level of TAZ and normal level of YAP (Figure 2A). These results demonstrate the specificity of our gene-editing approach in depleting YAP and TAZ proteins in EPCs. Both YAP-KD and TAZ-KD EPCs displayed cobblestone-like morphology (Figure 2B), expressed typical EPC surface markers, CD34, KDR, CD31, and CD144 (Figure 2C), and exhibited cytoskeletal organization similar to those of the unmanipulated EPCs (Figure 2D). These results suggest that depletion of YAP or TAZ did not affect the phenotypic characteristics of human EPCs.

### 3.3. Depletion of TAZ and YAP Caused Cell Cycle Arrest, Replicative Senescence, and Apoptosis in EPCs

**Figure 3:**
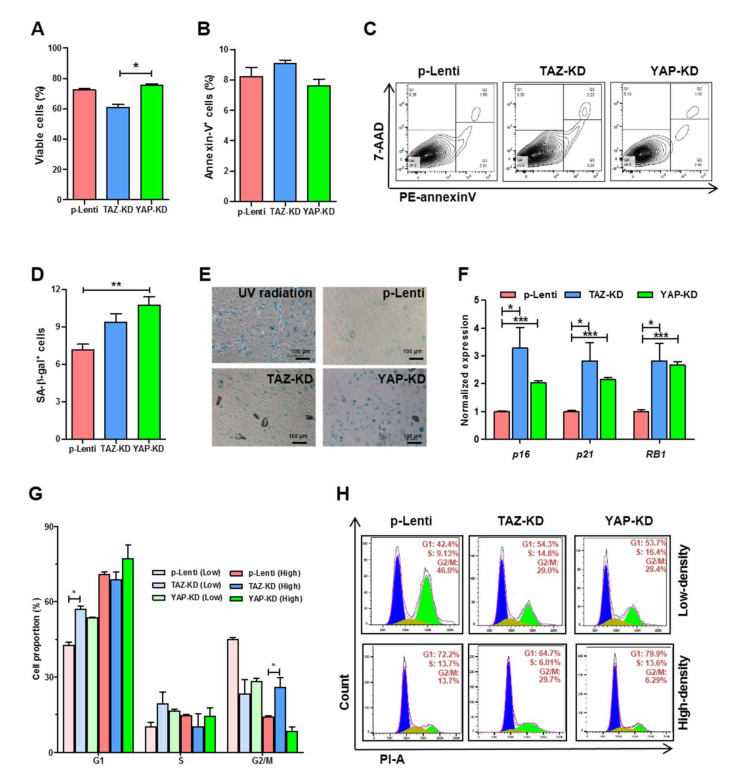
Survivability of YAP and TAZ-depleted EPCs. EPCs were synchronized by serum-starvation before seeding on fibronectin-coated wells before analyzing basal cell apoptosis (**A**–**C**) and β-galactosidase-positive senescent cells. 200 μM cisplatin was used as a positive control (**D**,**E**). Transcript levels of genes encoding cell cycle inhibitors; *p16*, *p21*, and *RB1* in control and KD EPCs were performed by RT-qPCR (**F**). Cell cycle analysis of control and KD-EPCs were determined by staining DNA contents with propidium iodide and analyzed by FACS analysis (**G**,**H**). All experiments were performed thrice independently with technical duplicates, and data were expressed as mean ± SEM. * *p*-value < 0.05; ** *p*-value < 0.01; and *** *p*-value < 0.001.

To determine the effects of YAP and TAZ on the proliferation and viability of EPCs, YAP-KD and TAZ-KD EPCs were subjected to cell cycle analysis, apoptosis assay, and b-galactosidase staining, which determine the number of senescent cells. TAZ-KD EPCs exhibited a lower level of cell viability and a higher level of apoptosis than controls. In contrast, YAP-KD EPCs showed the same levels of viability and apoptosis compared with controls (Figure 3A–C). Both TAZ-KD and YAP-KD EPCs have higher percentages of senescent cells in comparison to controls (Figure 3D,E). Upregulation of mRNA levels of cell cycle inhibitors *p16*, *p21*, and *RB1* in both TAZ-KD and YAP-KD cells confirms that the reduction of YAP and TAZ in EPCs caused replicative senescence (Figure 3F). Moreover, cell cycle analyses of EPCs at low- and high-cell density showed that TAZ depletion inhibited cell cycle progression of EPCs at the G1 phase in less confluent cells, while cell arrest at the G2/M phase was found in high-density cells (Figure 3G,H). These results suggest that TAZ is more important for EPC survival and proliferation than YAP.

### 3.4. Depletion of TAZ and YAP Caused Significant Impairment in EPC Functions

**Figure 4:**
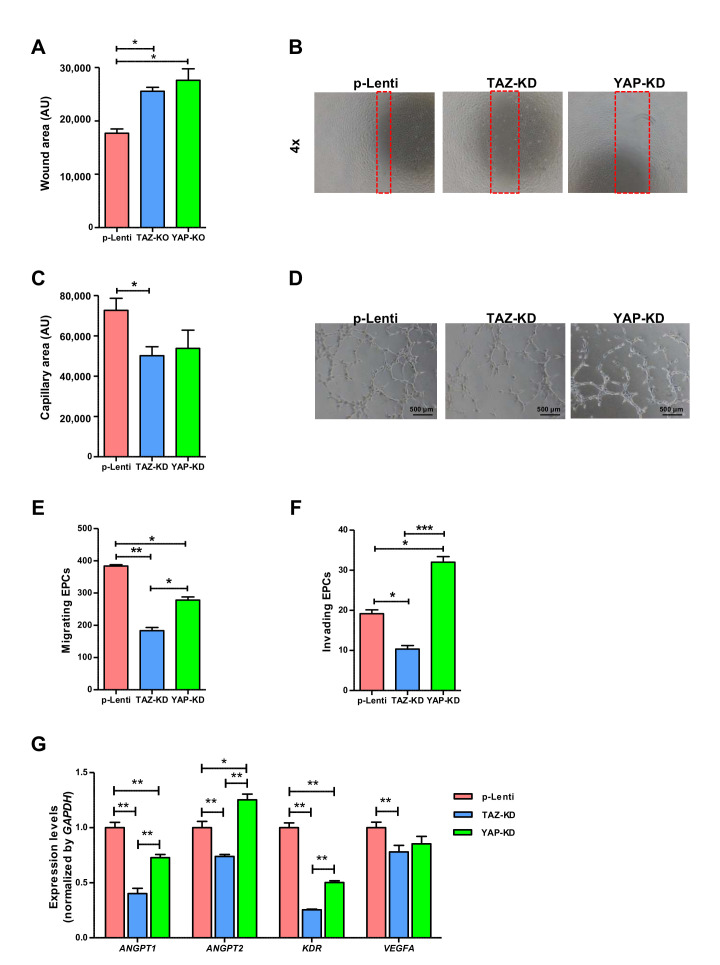
Endothelial cell function of YAP and TAZ-depleted EPCs. Synchronized EPCs were seeded on a fibronectin-coated plate for 24 h before performing wound-scratch assay using a 1-mm pipette tip width, and microscopic photos of wound closure were taken and quantified after 24 h (**A**,**B**). 3 × 10^4^ EPCs were seeded on undiluted Matrigel pre-coated wells of 4-well plate in a complete medium. Capillary-like structures were photographed at 12 h, and quantification of tube-formation area was assessed by ImageJ software (**C**,**D**). 5 × 10^4^ EPCs were plated on the upper chamber of Transwell in EndoGrow medium plus 2% FBS while lower chambers were filled up with complete medium plus 10% FBS, and cell migration and invasion were evaluated at 24 h post-incubation (**E**,**F**). RT-qPCR analysis for mRNA expression of endothelial cell-related genes (**G**). All experiments were performed three times independently with technical duplicates, and data were expressed as mean ± SEM. * *p*-value < 0.05; ** *p*-value < 0.01; and *** *p*-value < 0.001.

We further investigated the effects of YAP and TAZ on the migratory and vessel-forming capacity of EPCs which are critical for endothelial function. Depletion of TAZ significantly impaired the migratory capacity of EPCs in both scratch wound and transwell migration assays (Figure 4A,C,E). Moreover, TAZ-depleted EPCs have a lower ability to invade ECM and diminished vessel-forming capacity (Figure 4D,F). Similar to TAZ depletion, YAP depletion significantly impaired both migratory and vessel-forming capabilities of EPCs (Figure 4A,C,E). Contrary to TAZ depletion, YAP depletion enhanced EPC invasion instead of reducing it (Figure 4D,F). Similar to the effect of DH on endothelial gene expression, the depletion of YAP and TAZ down-regulated the expression levels of several endothelial genes, including *ANGPT1*, *ANGPT2*, *KDR*, and *VEGFA* (Figure 4G).

### 3.5. TAZ-KD EPC-Derived Conditioned Medium Diminished Stemness and Drug Resistance of Human Lung Cancer Cells

**Figure 5:**
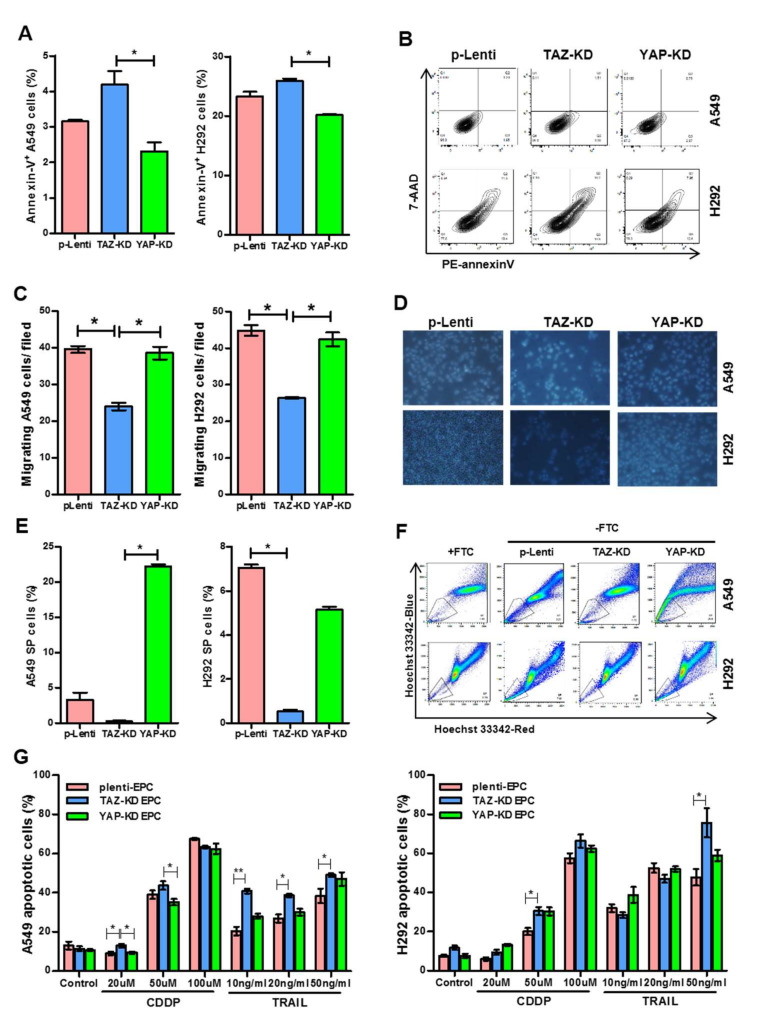
Treatment of lung cancer cell lines with conditioned medium from TAZ- or YAP-KD EPCs. Confluent NCI-A549 and H292 cell lines were exposed with a conditioned medium from EPCs for 24 h. Apoptosis detection (**A**,**B**), cell migration (**C**,**D**), and side population (**E**,**F**) were determined by annexin-V/7-AAD staining, Transwell assay, and Hoechst exclusion in the presence and absence of fumitremorgin C (FTC), respectively. A549 and H292 lung cancer cells cultured in an EPC-conditioned medium were treated with cisplatin (CDDP) and TRAIL, the apoptosis inducers, at indicated concentrations for 24 h. Apoptotic lung cancer cells were counted by Hoechst 33,342 staining of cells that have condensed or fragmented nuclei (**G**). All experiments were performed three times independently with technical duplicates, and data were expressed as mean ± SEM. * *p*-value < 0.05; and ** *p*-value < 0.01.

EPCs are known to promote cancer progression by releasing several cytokines such as monocyte chemotactic protein-1 [23] that enhance cancer survival, increase cancer migration, and induce tumor neovascularization. We, therefore, determined whether YAP/TAZ depletion affects this cancer-promoting property of EPCs by treating human lung cancer cell lines, A459, and H292 cells, with conditioned media derived from YAP-KD and TAZ-KD EPCs. Indeed, the conditioned medium derived from TAZ-KD EPCs increased apoptosis and reduced the migratory capacity of both H292 cells and A549 cells (Figure 5A–D). Moreover, the conditioned medium from TAZ-KD EPCs also reduced side population (SP) percentages, enriched for cancer stem cells, in both cell lines, and increased their sensitivity to chemotherapeutic agents cisplatin and TRAIL (Figure 5E–G). Distinct from TAZ-KD, the conditioned medium derived from YAP-KD EPCs did not affect the viability, migration, percentage of SP, and drug sensitivity of both H292 and A549 cells (Figure 5A–G).

### 3.6. VEGF Rescued the Capacity of EPCs to Form Tube-like Structure

**Figure 6:**
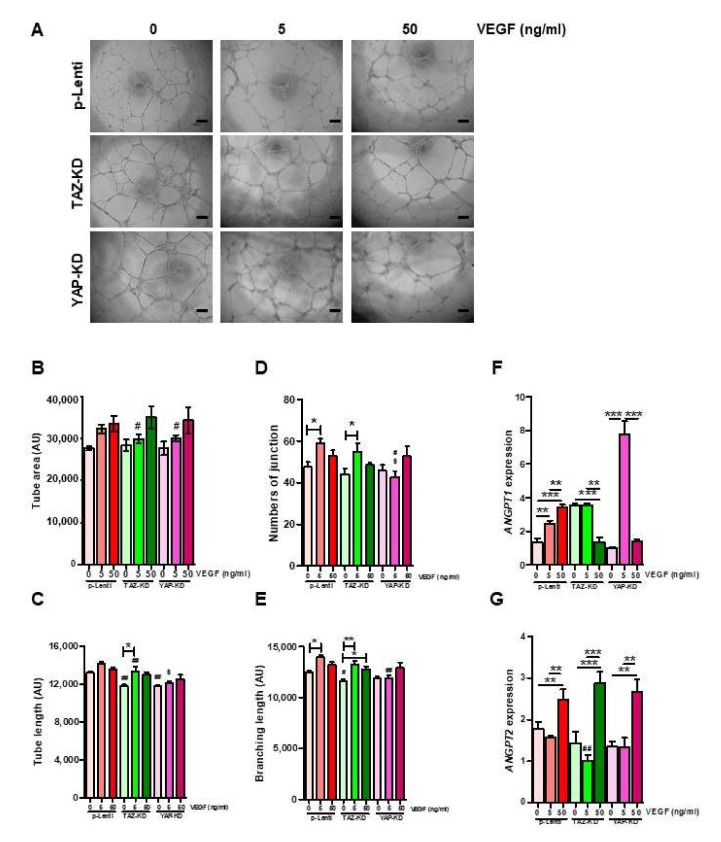
VEGF rescued tube-forming ability of KD-EPCs. 10,000 EPCs were seeded on Matrigel-coated wells of 96-well plate in quadruplicates and let to form the tube-like structure for 12 h in the absence or presence of VEGF at the indicated concentrations. Several angiogenic parameters were quantified by ImageJ software (**A**–**E**). The expression levels of ANGPT1 and ANGPT2 in the VEGF-treated EPCs were determined by qRT-PCR (**F**,**G**). * *p*-value < 0.05; ** *p*-value < 0.01; and *** *p*-value < 0.001; # *p*-value < 0.05, and ## *p*-value < 0.01 (TAZ-KD and YAP-KD vs. pLenti at the same dose of VEGF); ^$^ *p*-value < 0.05 (YAP-KD vs. TAZ-KD at the same dose of VEGF).

Impaired functions of YAP-KD and TAZ–KD EPCs might be caused by the down-regulation of VEGF expression in those cells. To test our hypothesis, YAP-KD and TAZ–KD EPCs were cultured in the presence of 5 and 50 ng/mL VEGF before being subjected to the tube formation assay. Addition of VEGF can rescue the ability to form the capillary-like structure of both YAP-KD and TAZ–KD EPCs as determined by several angiogenic parameters, including tube area, tube length, numbers of junction, and branching length (Figure 6A–E). Supplementation of VEGF at 50 ng/mL increased the expression of ANGPT2, but not ANGPT1 (Figure 6F,G).

### 3.7. Ectopic Expression of TAZ Restored the Impaired Functions of TAZ-Depleted EPCs

**Figure 7:**
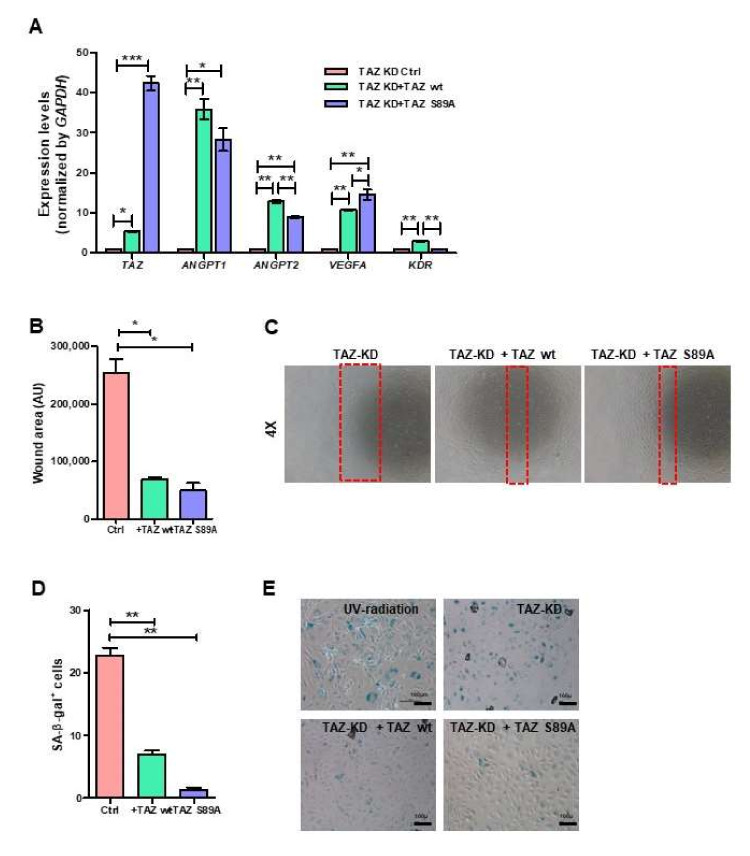
Rescue endothelial cell functions by ectopic expression of TAZ. TAZ-KD EPCs were nucleofected with wild-type or mutant S89A TAZ plasmids. mRNA of endothelial cell-related genes was quantified by RT-qPCR (**A**). Migration was measured by scratch-wound assay (**B**,**C**). Senescence cells were scored by enzymatic detection of positivity of β-galactosidase activity (**D**,**E**). All experiments were performed three times independently with technical duplicates, and data were expressed as mean ± SEM. * *p*-value < 0.05; ** *p*-value < 0.01; and *** *p*-value < 0.001.

To confirm the role of TAZ on the properties of EPCs, we rescued TAZ expression in TAZ-KD EPCs by overexpressing either wild-type TAZ or the constitutively active TAZ in TAZ-KD EPCs (TAZ-KD + wtTAZ and TAZ-KD + S89A-TAZ, respectively). The overexpression of both wild-type and constitutively active TAZ successfully restored TAZ expression in TAZ-KD EPCs (Figure 7A). Both TAZ-KD + wtTAZ and TAZ-KD + S89A-TAZ up-regulated the expression levels of *KDR*, *VEGFA*, *ANGPT1*, and *ANGPT2* in comparison to TAZ-KD control (Figure 7A). Moreover, the overexpression of wtTAZ and S89A TAZ also restored the migratory ability of TAZ-KD EPCs (Figure 7B,C) and reduced the percentages of senescent cells (Figure 7D,E).

## 4. Discussion

YAP and TAZ are related transcriptional co-activators that control mammals’ cell growth and organ size. YAP and TAZ regulate endothelial cells’ proliferation and metabolic activity during sprouting angiogenesis by up-regulating MYC [24]. YAP and TAZ also act as the central mediator of VEGF/KDR signaling to mediate the angiogenic response by activating a specific transcriptional program [25,26] and inducing migration of endothelial tip cells, which form new vessel branches, by activating small GTPase CDC42 [27]. In addition to angiogenesis, YAP/TAZ also plays a role in vascular rarefaction. This process removes excess vessels from the vascular network by regulating the expression of connective tissue growth factor (CTGF) and actin polymerization [28]. Depletion of WW-and-C2-domain-containing (WWC2), an upstream regulator of the Hippo signaling pathway and inhibitor of YAP/TAZ, leads to the hyperproliferation of endothelial cells with enlarged sprouting areas and increased tip cell numbers in the ocular vessels of postnatal mice [8].

Apart from mature endothelial cells, there is increasing evidence that the circulating endothelial progenitor cells (EPCs) play essential roles in tissue neovascularization by releasing several pro-angiogenic cytokines and generating new functional endothelial cells. However, the roles of YAP/TAZ in regulating EPC functions are still largely unknown. Therefore, in the present study, we investigate whether the depletion of YAP/TAZ in primary human EPCs causes EPC dysfunction. Firstly, we inhibited the activation of YAP/TAZ using a small molecule inhibitor dobutamine (DH) and found that DH reduced the level of active YAP protein and significantly downregulated the expression levels of *VEGFA* and *KDR* (VEGF receptor) genes. These results confirmed that DH inhibits YAP activity. Reduction of YAP impaired the ability of EPCs to form the capillary-like structure on Matrigel (Figure 1F,G) and induced cell cycle arrest of DH-treated EPCs at the S-phase (Figure 1H,I).

We then specifically depleted YAP or TAZ expression in EPCs using CRISPR/Cas9 and showed that the depletion of either YAP or TAZ did not significantly alter the morphology and surface marker expression profile of EPCs. Regarding the EPC functions, our results show that TAZ depletion negatively affected migratory, invasive, and vessel-forming capacities of EPCs. Moreover, TAZ depletion also reduced EPC viability, inhibited cell cycle progression, and increased EPC senescence. Like TAZ depletion, YAP depletion also increased EPC senescence, reduced migration, and hampered their ability to form capillary-like structures. However, YAP depletion did not affect EPC viability and enhanced their invasion instead of inhibiting it.

We further addressed the effect of YAP and TAZ on the endothelial gene as YAP and TAZ serve as transcriptional co-activators that interact with TEAD to control the transcription of several endothelial genes. We found that the depletion of YAP/TAZ downregulated the expression levels of several pro-angiogenic genes, including *ANGPT1*, *ANGPT2*, *VEGFA*, and *KDR* that have been shown to play critical roles in regulating EPC functions and the neovascularization process. The downregulation of those endothelial genes might be responsible for the negative effects of TAZ/YAP depletion on the EPC functions.

Although the redundant role of YAP and TAZ has been suggested in several contexts, their roles in human EPCs have yet to be elucidated. Herein, we showed that YAP and TAZ are essential for EPC survival, migration, invasion, and differentiation. However, TAZ seems to be more crucial for EPC functions as TAZ depletion induces more defective phenotypes in EPCs than the YAP depletion. Considering all results obtained in this study, it is also possible that YAP and TAZ play distinct roles in regulating EPC activity and endothelial-related gene expression.

To further confirm the roles of TAZ on EPC functions, we determined whether the overexpression of wild-type and constitutively active TAZ can rescue the defective functions of TAZ-KD EPCs. Indeed, the overexpression of wild-type and constitutively active TAZ increased the expression levels of endothelial genes, restored the migratory capacity, and reduced the senescence of TAZ-KD EPCs.

EPCs have been known to promote cancer progression by releasing several cytokines that induce tumor neovascularization and enhance survivability and migratory capacity of cancer cells [29,30]. We showed that the conditioned medium derived from TAZ-KD EPCs increased apoptosis, inhibited migration, reduced the percentage of side population (SP) enriched for cancer stem cells in human lung adenocarcinoma cell lines, and increased their sensitivity to chemotherapeutic agents. These results suggest that TAZ depletion inhibited the tumor-promoting property of EPCs. Distinct from TAZ-KD, the conditioned medium derived from YAP-KD EPCs did not affect the viability, migration, percentage of SP, and drug sensitivity of human lung adenocarcinoma cells.

The underlying mechanism is essential for understanding the roles of YAP and TAZ in regulating EPC functions. Our results demonstrate that the reduction of YAP and TAZ decreased the expression level of VEGF in EPCs. VEGF has been shown to play a central role in regulating the proliferation, migration, and vessel- formation of both endothelial cells and EPCs. We presumed that the impaired functions of YAP-KD and TAZ–KD EPCs might be a consequence of the down-regulation of VEGF expression in those cells. To prove this, YAP-KD and TAZ–KD EPCs were cultured in the presence of 5 and 50 ng/mL VEGF before being subjected to the tube formation assay. The results support our hypothesis by showing that the addition of VEGF can rescue the ability to form the capillary-like structure of both YAP-KD and TAZ–KD cells, and the effect of VEGF on the vessel-forming capacity of both YAP-KD and TAZ–KD EPCs seem to be dose-dependent manner. The addition of 50 ng/mL VEGF also increased the expression level of the *ANGPT2* gene, which is down-regulated in both YAP- and TAZ–KD cells. The previous study showed that ANGPT2 secreted from endothelial cells regulates the angiogenesis and vascular remodeling processes by activating the endothelial TIE2 receptor in an autocrine manner [31]. Taken together, our additional results suggest that YAP and TAZ regulate EPC function by up- regulating the expression of VEGF. The increased level of VEGF, in turn, enhances the vessel-forming capacity of EPCs and increases the expression of *ANGPT2*, which also plays an essential role in angiogenesis. To validate our proposed mechanism, further in vivo studies will be needed.

These results suggest that although YAP and TAZ are considered to have similar functions, there is a distinction between the effects of YAP and TAZ on the invasive capacity and tumor-promoting property of EPCs. These results agree with the previous study, which showed that YAP and TAZ have distinct cellular functions in a context-dependence manner [32,33]. Unfortunately, the excessive cell death observed in our double YAP and TAZ-knockdown (YAP/TAZ-KD) EPCs makes it impossible to directly compare the effect of single YAP or TAZ depletion with double depletions of YAP/TAZ in this study.

Taken together, we herein demonstrated the distinct effects of TAZ and YAP on the biological properties of primary human EPCs. Modulating YAP and TAZ activities could benefit patients with EPC dysfunction, such as patients suffering from diabetes mellitus or other vascular diseases, to enhance EPC functions and promote neovascularization in their ischemic or injured tissues [34,35]. Moreover, the inhibition of TAZ might also be used to inhibit the tumor-promoting effect of EPCs and prevent tumor progression in cancer patients.

## Figures and Tables

**Table 1:** Primer sequences used in RT-qPCR.

Gene Name	Forward (5′ → 3′)	Reverse (5′ → 3′)
*YAP*	CAGGTTGGGAGATGGCAAAG	TGTTGTCTGATCGATGTGATTTAAGA
*TAZ*	CAATAGCTCAGATCCTTTCCT	TAGTATCACCTGTATCCATCTC
*VEGFA*	TCCTCACACCATTGAAACCA	GATCCTGCCCTGTCTCTCTG
*KDR*	CTGGCATGGTCTTCTGTGAA	TTCCATGAGACGGACTAGA
*ANGPT1*	AGGAACCAGCCTCCTCTCTC	TTCTCCAGCAGCTGTATCTCAA
*ANGPT2*	AACCAGACGGCTGTGATGAT	TTGTCGAGAGGGAGTGTTCC
*p16*	TCGTGCTGSTGCTACTGAGG	ACCAGCGTGTCCAGGAAG
*p21*	GGATTAGGGCTTCCTCTTGG	TTTAGCAACAGTGGGGTCCT
*RB1*	GCAAATGCAGAGACACAAGC	CTGGAAAAGGGTCCAGATGA
*GAPDH*	GTCAACGGATTTGGTCGTATTG	CATGGGTGGAATCATATTGGAA

## Supplementary Materials

The following are available online at https://www.mdpi.com/article/10.3390/biomedicines10010147/s1. Figure S1: Nucleo-cytoplasmic fractionation and immunoblotting to determine the levels of non-phosphorylated and phosphorylated YAP/TAZ proteins in the DH-treated EPCs.
