# Supplementary material for: Distinctive Roles of YAP and TAZ in Human Endothelial Progenitor Cells Growth and Functions
Source: Biomedicines. 2022 Jan 11;10(1):147. doi: 10.3390/biomedicines10010147 (PMC8773510; doi:10.3390/biomedicines10010147)
Supplement: Supplementary file 1 [file biomedicines-10-00147-s001.zip › biomedicines-1471940-supplementary.pdf]

Sup Fig 1

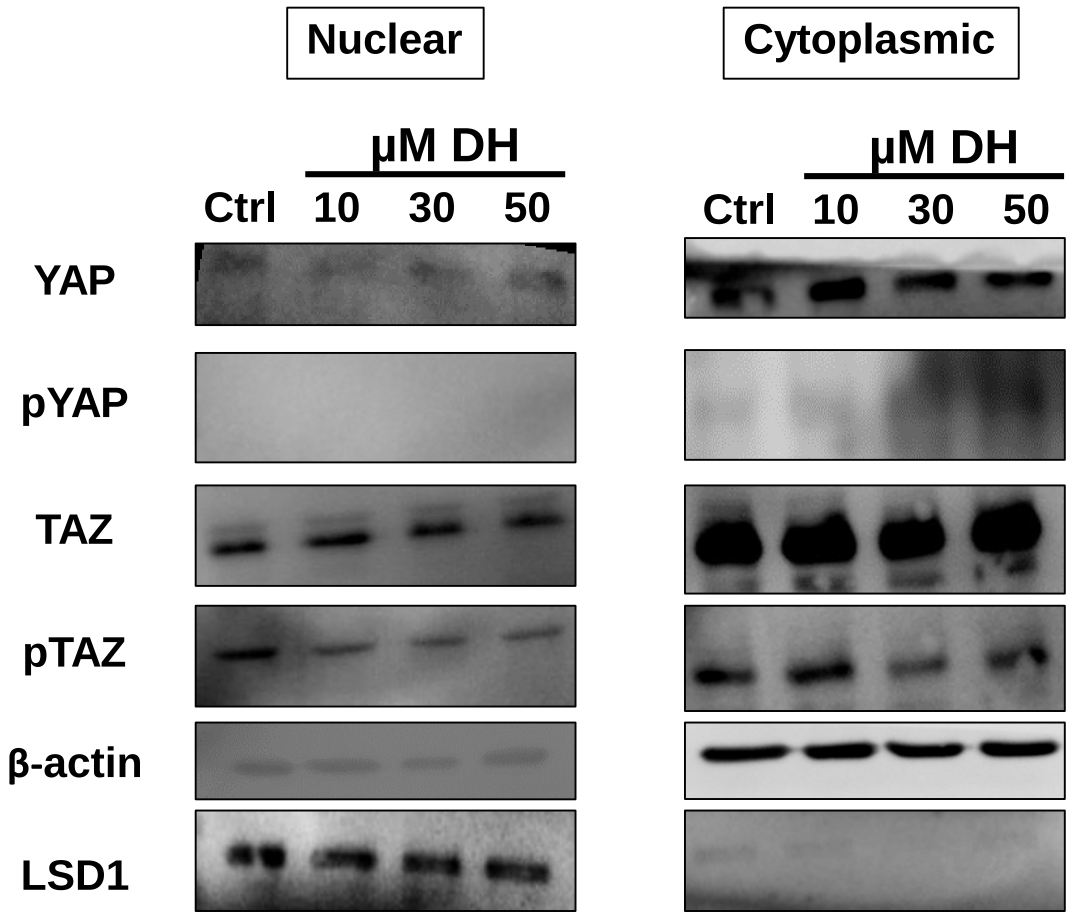

Supplementary Figure S1. Nucleo-cytoplasmic fractionation and immunoblotting to determine the levels of non-phosphorylated and phosphorylated YAP/TAZ proteins in the DH-treated EPCs.
